# By-Products of Agri-Food Industry as Tannin-Rich Sources: A Review of Tannins’ Biological Activities and Their Potential for Valorization

**DOI:** 10.3390/foods10010137

**Published:** 2021-01-11

**Authors:** María Fraga-Corral, Paz Otero, Javier Echave, Paula Garcia-Oliveira, Maria Carpena, Amira Jarboui, Bernabé Nuñez-Estevez, Jesus Simal-Gandara, Miguel A. Prieto

**Affiliations:** 1 Nutrition and Bromatology Group, Analytical and Food Chemistry Department, Faculty of Food Science and Technology, Ourense Campus, University of Vigo, 32004 Ourense, Spain; mfraga@uvigo.es (M.F.-C.); pazoterofuertes@gmail.com (P.O.); ecalja@outlook.es (J.E.); paula.garcia.oliveira@uvigo.es (P.G.-O.); maria.carpena.rodriguez@uvigo.es (M.C.); jarb.amira@gmail.com (A.J.); bernabenunez16@gmail.com (B.N.-E.); 2 Centro de Investigação de Montanha (CIMO), Campus de Santa Apolonia, Instituto Politécnico de Bragança, 5300-253 Bragança, Portugal; 3 Department of Pharmacology, Pharmacy and Pharmaceutical Technology, Faculty of Veterinary, University of Santiago of Compostela, 27002 Lugo, Spain

**Keywords:** tannins, valorization, circular economy, biological properties, health benefits

## Abstract

During recent decades, consumers have been continuously moving towards the substitution of synthetic ingredients of the food industry by natural products, obtained from vegetal, animal or microbial sources. Additionally, a circular economy has been proposed as the most efficient production system since it allows for reducing and reutilizing different wastes. Current agriculture is responsible for producing high quantities of organic agricultural waste (e.g., discarded fruits and vegetables, peels, leaves, seeds or forestall residues), that usually ends up underutilized and accumulated, causing environmental problems. Interestingly, these agri-food by-products are potential sources of valuable bioactive molecules such as tannins. Tannins are phenolic compounds, secondary metabolites of plants widespread in terrestrial and aquatic natural environments. As they can be found in plenty of plants and herbs, they have been traditionally used for medicinal and other purposes, such as the leather industry. This fact is explained by the fact that they exert plenty of different biological activities and, thus, they entail a great potential to be used in the food, nutraceutical and pharmaceutical industry. Consequently, this review article is directed towards the description of the biological activities exerted by tannins as they could be further extracted from by-products of the agri-food industry to produce high-added-value products.

## 1. Introduction

### 1.1. Tannins as Target Compounds

**Figure 1:**
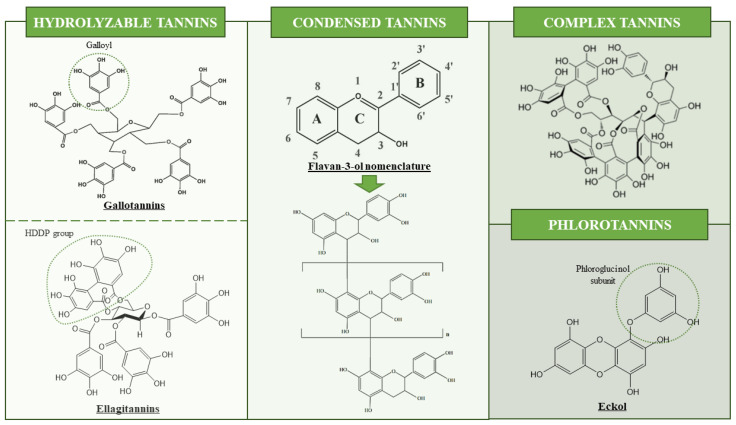
Structural classification of tannins. Functional groups are shown in circles.

Tannins are a diverse group within phenolic compounds widely distributed in nature. They are secondary metabolites of plants usually produced as a result of stress and they exert a protective role, including photoprotection against UV rays and free radicals or defense against other organisms and environmental conditions, such as dryness [1,2,3]. Tannins are a heterogeneous group, having molecular weights between 500 and 20,000 Da and very different chemical structures [4]. Tannins have been demonstrated to exert different biological activities, such as antioxidant activity. This property is related to their chemical structure as they possess phenolic rings able to bind to a wide range of molecules and act as electron scavengers to trap ions and radicals [2,4]. Generally, tannins possess about 12–16 phenolic groups and five to seven aromatic rings per 1000 Da [5]. They also present plenty of hydroxyl groups, which confer on them hydrophilic properties, solubility in aqueous solvents and also the ability to form complexes with proteins, carbohydrates, nucleic acids and alkaloids [6,7]. Regarding tannin classification, they have been historically classified into hydrolyzable tannins (HTs) and condensed tannins (CTs), and the latter are also called proanthocyanidins. Nowadays, the classification according to their chemical characteristic and structural properties has been updated. Thus, tannins can be grouped into gallotannins, ellagitannins, CTs, complex tannins (CoTs) and phlorotannins (PTs, an exclusive class of tannins found in the algal species of the Phaeophyceae class) [1,2,8]. A schematic representation of tannin structural classification is presented in Figure 1.

HTs have owned their name as they can be hydrolyzed by weak acids/bases, producing carbohydrates and phenolic acids because of the reaction [3]. They are formed by glycosylated gallic acid units [9], which can be either ellagic acid (EA) or gallic acid (GA), forming ellagitannins (ETs) and gallotannins (GTs), respectively [3,10]. ETs are formed by simple to multiple units of hexahydroxydiphenol (HHDP) connected to a polyol core. After hydrolysis and the breakdown of C-C bonds between suitably orientated galloyl residues of glucogalloyl molecules of HHDP, they are converted into EA units [2,11,12]. The abundant variety of structure has been observed within this group, due to the different possibilities in the formation of oxidative linkages [7]. On the other hand, GTs are considered as simpler HTs and are formed by galloyl or digalloyl units coupled to a polyol, catechin or triterpenoid unit, in the form of pentagalloyl glucose (PGG). They can yield GA from the hydrolysis reaction [2,13]. HTs can be mainly found in fruits, berries, legumes, leafy vegetables and different tree species [3,9]. They have been widely employed in the leather industry and they have been studied for their antioxidant and antimicrobial properties [3].

CTs or proanthocyanidins account for more than 90% of the world commercial production of tannins [3]. They are polymeric or oligomeric flavan-3-ols, formed by the combination of A (phloroglucinol or resorcinol) and B (catechol or pyrogallol) rings [2,14] (Figure 1). When these compounds are heated in ethanol solutions in acidic conditions, they are decomposed into anthocyanidins [7]. The combination of these flavan-3-ol monomers gives rise to the formation of procyanidins (PCs) (composed of catequins and epicatechins) or profisetinidin, prorobinetidin and prodelphindin (composed of (epi)fisetinidol, (epi)robinetinidol and (epi)gallocatechin units) [2,15]. Particularly, these type of tannins are commonly found in fruits, berries, cocoa and some drinks such as wine, beer or tea [9].

Finally, CoTs and PTs will be briefly described. CoTs are tannins of high molecular weight, created as a result of the bonding between flavan-3-ols with either GTs or ETs. This type of tannin can be obtained from tree species such as *Quercus* sp. and *Castanea sativa* [6]. Finally, PTs are polyphenols obtained from brown marine algae and formed by phloroglucinol (1,3,5-trihydroxybenzene) (PG) synthesized via the acetate–malonate pathway. They are grouped into six major groups (fucols, phloroetols, fucophloroteols, fuhalols, carmalols and eckols) depending on to the type of bonds between PG units and their content of hydroxyl groups, being more complex with a higher level of PG units [16,17]. These tannins, which can represent up to 30% of seaweeds’ dry weight, have been demonstrated to exert antimicrobial, photoprotection or antioxidant activities, among others [17,18].

Although tannins have been sometimes linked to unpleasant organoleptic properties, they have also shown plenty of properties and applications. Some of these properties are antioxidant, antimicrobial or anti-inflammatory, among others, which have given rise to their use in the food, nutraceutical and pharmaceutical industry [19]. Additionally, their toxic effects have been assessed [1]. Particularly, they have been proposed as natural food additives able to enhance the safety and the shelf life of products and also as clarification agents in drinks [20]. Furthermore, tannins have been used as adhesives and coatings, foams or adsorbents, among many other applications [3]. Different species containing diverse tannins have been included as part of patents that aim to exploit their properties and to create innovative applications (Table 1).

### 1.2. Circular Economy and Exploitation of By-Products

In this context, the wide variety of biological activities exerted by tannins and their natural sources are the perfect scenario for the implementation of a valorization strategy. In recent decades, natural products and ingredients have gained an increasing demand instead of the use of synthetic additives. Consumers opt for this option as they are safer, ecofriendly and they show plenty of health benefits, while avoiding side effects associated with synthetic antimicrobials [21,22]. Additionally, the concept of a circular economy has been boosted in recent years, whose principal idea relies on closing loops, creating complete cycles of production [23]. Several studies have suggested that the food production system could join the circular economy model by adapting its manufacture model and valorizing by-products of the agri-food industry [24]. Hence, considering that tannins are widely distributed among vegetation of the terrestrial and aquatic environments and the disposal of by-products of the agri-food industry such as leaves, peels or seeds that can be used as tannin-rich sources, valorization of tannin recovery might be a feasible approach [2,25,26]. Besides, brown algae are also potential sources of tannins as they are easily harvested, sometimes underutilized and, in other cases, considered as invasive species and, thus, their elimination from the environment is advisable [27]. The presence of tannins in agricultural wastes opens the possibility to obtain them from sustainable and affordable sources. This sustainable approach must further be supported by the continuous search for and optimization of novel extraction methods (i.e., solid liquid, ultrasound, microwave, supercritical fluids or high-pressure extraction) together with the use of green solvents [2,28]. Then, obtaining added-value products from underutilized by-products contributes to the development of a circular economy. Altogether, these measures would contribute to lowering the environmental impact of human activity, generate new and affordable added-value products and reduce the economic cost of recycling and waste management [29]. Yet, waste derived from tannin-containing plants can be used to extract these valuable products, with multiple potential applications [30].

Considering the aforementioned properties of tannins and their availability in nature, this review article is aimed at compiling the main biological activities of tannin-rich extracts to evaluate their potential use for food, nutraceutical and pharmaceutic applications, valorizing by-products of the agri-food industry as potential sources to produce added-value tannin-based products.

## 2. Biological Activities of Tannin-Rich Extracts

As aforementioned, tannins represent a chemical defense barrier for plants and algae that improve the response against pathological attacks and adverse abiotic conditions. The biological activity in plants and algae has prompted their utilization as traditional remedies to treat numerous diseases or infections. Currently, the biological effects of purified tannins or tannin-rich extracts (containing additional biomolecules) have been evaluated in vitro and in vivo using animal models, and more recently by clinical trials performed on humans [31]. Most of these research works have been focused on the study of the bioactivities of plants containing high amounts of tannins or, less commonly, purified tannins, to disclose their potential for developing innovative applications in the field of medicine, pharmacology, cosmetics, botany and/or veterinary medicine [10]. Among the biological activities of tannins, the most relevant ones are antioxidant, anti-inflammatory, anti-diabetic, cardioprotective, healing and antimicrobial (antiviral and antibacterial) [10,32] (Table 2).

### 2.1. Antioxidant

Tannins, as other polyphenols, present the ability to scavenge diverse free radicals and inhibit lipid peroxidation. In fact, their content increases under stressful conditions in cellular pro-oxidant states. This activity is related to the presence of phenolic rings in the chemical structure of the compound and also the degree of polymerization [2,70].

The application of tannins as antioxidants has been evaluated in living cells. For example, tannic acid is commonly utilized as purified tannin. Its antioxidant capacity has been demonstrated in an in vitro assay based on fibroblasts irradiated with UVB to create an oxidative environment that led to cellular damage, simulating photoaging. Tannic acid showed strong antioxidant properties, a broad UV absorption spectrum and also an inhibitory activity towards collagenase and elastase. Thus, this tannin acid was demonstrated to prevent photodamage by attenuating the evaluated oxidation levels and diminished photoaging parameters [71]. The antioxidant activity of many other types of tannins has been evaluated. In a previous study, the ability to prevent lipid peroxidation of diverse phenolic compounds, including 25 tannins (both CTs and HTs at 5 µg/L), was assessed in rat liver mitochondria. The results displayed that HTs, like pedunculagin, PGG and chebulinic acid, were the most effective inhibitors. This suggest that the presence of structures such a galloyl, HHDP or dehydro-HHDP are involved in the inhibition of lipid peroxidation [72]. Similar results were observed when different tannins (CTs: catechin and epigallocatechin-gallate; HTs: PGG and geraniin) were used to inhibit the effects of the induced lipid peroxidation in mouse lens [73]. The seed coat from *Phaseolus vulgaris* was used to extract and purify tannins and flavonoids and the antioxidant activity of these compounds was tested using a liposome assay. Delphinidin and petunidin-3-glucoside were the most active tannins, showing an activity close to the 50% of that of the synthetic antioxidant butylated hydroxytoluene (BHT) [74]. Another work characterized a CT form from *Diospyros kaki* and then analyzed its antioxidant capacity by an ex vivo tissue system and an in vivo assay. When tested in a mouse liver homogenate, it showed strong protection against auto-oxidation and H_2_O_2_-induced oxidation processes with half inhibition concentrations of 4.3 and 1.4 μg/mL, respectively. In vivo assays, performed by the oral administration of 200 or 400 mg of CT per kg of mouse body weight, displayed a reduction of the activities of the evaluated oxidative biomarkers (serum and liver superoxide dismutase (SOD), GSH (reduced glutathione) peroxidase and liver malondialdehyde (MAD) activities) [75]. The antioxidant properties of tannin-rich extracts obtained from different vegetal species have been assessed. For instance, a *Q. robur* tannin-rich extract, fundamentally composed of roburins, castalagin and vescalagin, has been reported to ameliorate oxidative stress markers and serum levels of related enzymes such as catalase (CAT) or SOD in a clinical trial [56]. Another trial with the same extract studied the effect in vivo and ex vivo, analyzing the plasmatic oxidative profile and genetic expression of cell cycle-related genes from plasma cell samples and several tissues. Although the study sample consisted of only three subjects, the results were significant and very similar in all three subjects, with a significant increase in phenolic concentration in plasma, as well as the modulation of targeted genes [57]. A study evaluated an extract from *A. mearnsii,* which displayed antioxidant properties that reversed the negative effects caused by acrolein (a compound related to neurodegenerative diseases)-induced cytotoxicity in a human neuroblastoma cell line (SH-SY5Y). In addition, the extract also inhibited the action of apoptotic factors [76]. In the same line of experiments, oxidative stress was induced in the SH-SY5Y cell line after its previous treatment with *C. sativa* extracts, which significantly reduced reactive oxygen species (ROS) production. In addition, it was observed that the previous treatment reduced apoptotic signals caused by the damage inducers [37].

**Figure 2:**
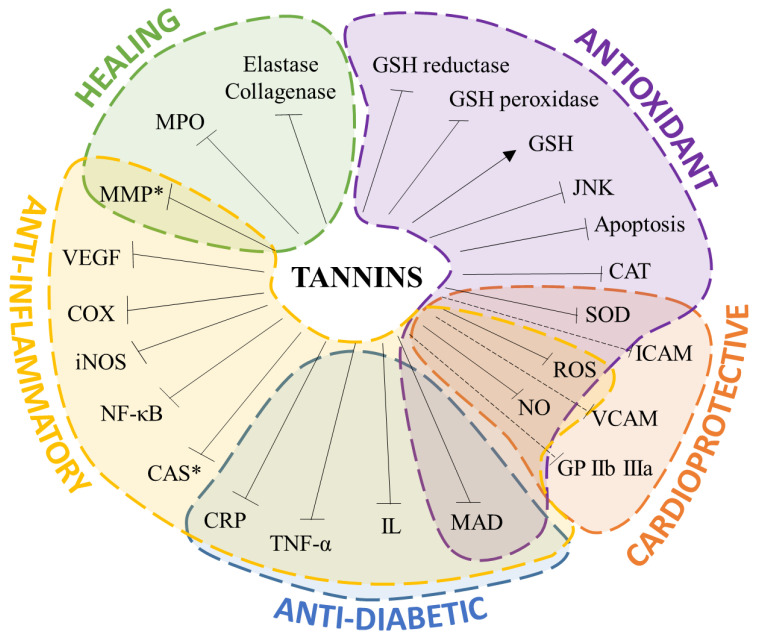
Visual representation of the suggested mechanisms involved in the biological properties of tannins. Lines show decrease in or inhibition of biomarkers, whereas arrows show an increase in or promotion of reduced glutathione (GSH). (* = antioxidant activity; MAD: malondialdehyde; IL: interleukin; TNF-α: tumor necrosis factor-α; CRP: c-reactive protein; CAS: caspase; NF-κB: nuclear factor-κB; iNOS: nitric oxide synthase; COX: cyclooxygenase; VEGF: vascular endothelial growth factor; MMP: matrix metalloproteinase; JNK: C-Jun N-terminal kinase; MPO: myeloperoxidase; CAT: catalase; SOD: superoxide dismutase; ROS: reactive oxygen species; NO: nitric oxide; VCAM: vascular cell adhesion protein; ICAM: intercellular adhesion molecule; GP IIb IIIa: glycoprotein IIb/IIIa).

Although the antioxidant mechanism of tannins has been repeatedly investigated, deeper studies about the concrete mechanism of action are needed, especially considering the administration as well as the variability of the tannin metabolic profile associated with each species. Additionally, it is worth mentioning that the antioxidant capacity is the basis for triggering further systematic and beneficial effects, such as anti-inflammatory responses and wound healing (Figure 2).

### 2.2. Anti-Inflammatory

Recently, numerous works have disclosed the mechanism of action of the anti-inflammatory effect of several tannins. However, many other works demonstrate the systemic effects of these natural molecules without presenting the specific cellular mechanism or without identifying the specific compounds responsible for the effect. We have tried to focus on those providing the chemical profile and mechanism of action.

An in vivo assay using mice applied an aqueous extract from the bark of *A. nilotica* by intraperitoneal injection to determine its antinociceptive, anti-inflammatory and antipyretic activity. The extract results displayed a slight reduction in paw edema. In addition, *Acacia* treatment (150 mg/kg body weight) was able to inhibit the formalin-induced inflammation at values like those of diclofenac sodium. The antipyretic treatment was maintained for 3 h and showed a slight inhibitory effect on fever after inducing the pyrexia with yeast [36]. Another study, using in vitro techniques, analyzed different extracts from leaves of *A. mearnsii.* The most active one possessed at least (epi)fisetinidol derivatives (with catechin, gallocatechin, additional molecules of fisetinidol or even robinetinidol) quantified at 12.6 mg/g as procyanidin B2 equivalents. This fraction, applied at non-cytotoxic levels (50 μg/mL) in RAW 264.7 macrophages, previously exposed to oxidative stress, was able to significantly inhibit ROS production and reduce nitric oxide (NO) back to non-stimulated levels. Later, the same cell culture was exposed to an inflammatory process through lipopolysaccharide (LPS) stimulation. This assay showed that the *A. mearnsii* extract inhibited the expression of cytokines like interleukin-1β or -6 (IL-1β, IL-6) and pro-inflammatory enzymes such as cyclooxygenase-2 (COX-2) or inducible nitric oxide synthase (iNOS) [33]. Similar results were observed in LPS-stimulated RAW 264.7 macrophages treated with a *T. chebula* extract. Among the identified molecules, two GTs (chebulinic acid and 2,3,6-tri-O-galloyl-β-D-glucose) applied at 50 µM could reduce NO production and decreasing the protein expression of iNOS and COX-2 [68]. The anti-inflammatory properties of extracts from the spiny burs of *C. sativa* have also been tested in vitro using LPS induction in the BV-2 cell line, simulating a microglia model. The treatment showed cytoprotection of the downregulation of the expression of IL-1β, tumor necrosis factor-α (TNF-α) and nuclear factor-κB (NF-κB) [38]. Following similar approaches, an inflammatory process was induced in the HaCaT cell line using tumor necrosis factor-α (TNF-α). Then, the cells were treated with ethanolic extracts from *R. coriaria* obtained by maceration or cold extraction. The induction with TNF-α stimulates pro-inflammatory signals by the production of interleukins, vascular endothelial growth factor (VEGF), matrix metallopeptidase 9 (MMP-9) and intercellular adhesion molecule 1 (ICAM-1). This inflammatory cascade was inhibited by both kinds of *R. coriaria* extracts, except for VEGF, which was just decreased by the maceration extract [58]. Another study, performed in vivo, orally administrated *R. coriaria* extracts to rats to study its ability to prevent or treat necrotizing enterocolitis. The antioxidant, anti-inflammatory, immunomodulatory and antiapoptotic abilities of *R. coriaria* were analyzed through the quantification of oxidative indicators and histological assays. The application of the treatment reduced the presence of inflammatory molecules in histological samples, while biochemical results reported lower amounts of IL-6, TNF-α and lipid hyperoxides. Besides, the negative effects of induced necrotizing enterocolitis were reversed [59]. In another in vivo study using rats, the ability of procyanidins obtained from grape seeds to reduce inflammation induced by a hyperlipidic diet was analyzed. The oral administration of these tannins produced a down-regulation of C-reactive protein (CRP), TNF-α and IL-6 in liver and white adipose tissue [77]. However, in a comparative work of extracts from *R. occidentalis* and *Vitis labrusca* seeds, the analysis of the former showed higher contents of tannins and also stronger antioxidant and anti-inflammatory properties [78]. Indeed, many works have been carried out using, as a basis, species belonging to the genus *Rubus* to show their potential bioactivities. For instance, an extract of *R. fruticosus* was evaluated as an antioxidant, anti-inflammatory and gastroprotective agent in rats. The anti-inflammatory effects reported by the histological exam were attributed to cyanidin-3-glucoside through the reduction or inhibition of the activity of NF-κB, COX-1 and -2, NO and/or iNOS [60]. In vitro assays using another species of the same genus, *R. idaeus*, demonstrated the reductio of inflammation and oxidation in hypertrophied adipocytes. Extracts from fruits of *R. idaeus* were able to down-regulate the expression of IL-1β and -6, TNF-α and leptin but also up-regulate the expression of antioxidant enzymes, such as SOD and CAT. Apart from these main mechanisms, the application of *R. idaeus* extracts reduced lipid accumulation and increased lipid mobilization in hypertrophied adipocytes, which may help to prevent the future appearance of further metabolic disorders [79].

As shown in these previous works, antioxidant and anti-inflammatory activities of tannins can have positive collateral effects. Therefore, tannins have been tested as natural ingredients with preventive or treatment purposes in many diseases or infections whose main bases are oxidative and inflammatory processes such as diabetes, heart infections or wound healing.

### 2.3. Antidiabetic

In a recent in vivo experiment performed in rats, the efficacy of *R. fruticosus* as a source of natural antidiabetic agents was supported. hydroethanolic extracts of *R. fruticosus* were administered by intraperitoneal injection to streptozotocin-induced diabetic rats. Diabetes, like many other chronic diseases, has been found to trigger oxidative and inflammatory processes at a cellular level. The intraperitoneal administration of *R. fruticosus* was demonstrated to reduce oxidative and inflammatory markers such as TNF-α, IL-6 and CRP [61]. Different species recognized as tannin-rich sources have been also described as potential sources of antidiabetic agents, such as *C. sativa. Q. robur, S. lorentzii* or *T. chebula*. Tannins, especially HTs, have been reported to inhibit α-glucosidase, an enzyme responsible for the absorption of carbohydrates from the gut. In fact, inhibitors of α-glucosidase may be used in the treatment of patients with type 2 diabetes mellitus (DM) or impaired glucose tolerance [39]. Therefore, the administration of tannins for the prevention or treatment of type 2 DM may have a doubly positive effect, as an antioxidant and as an α-glucosidase inhibitor. For instance, different extracts from the wood of *C. sativa* were tested as potential α-glucosidase inhibitors and as antioxidants. An initial extract was fractioned into five parts, from which the best one was fractioned into seven parts. From these seven fractions, the ones with the strongest antioxidant capacity were mostly composed of the phenolic acid GA, grandinin, valoneic acid dilactone and its galloyl derivative and trigalloylglucose (TGG) molecules. The extracts with stronger α-glucosidase inhibition contained valoneic acid dilactone, three TGG isomers and PGG. The molecule common to these two extracts was the valoneic acid dilactone that has previously been reported as an α-glucosidase inhibitor [39]. Similarly, from *Q. robur*, different fractions were tested for antioxidant and α-glucosidase inhibition activities, with the molecules involved in the strongest antioxidant role being a monogalloylglucose (MGG) isomer, an HHDP-glucose isomer, castalin, GA, vescalagin and grandinin/roburin E isomer. The sub-fraction with the strongest α-glucosidase inhibitory activity contained castalagin as the major tannin [55]. From *S. lorentzii*, fractions composed of HTs, esters of quinic acid with different units of GA (di-, tri-, tetra- and penta-galloylquinic acids) and oligomeric CTs (dimers, trimers, tetramers or pentamers of catechin or catechin-3-O-gallate and fisetinidol, or catechin-3-O-gallate) possessed the strongest α-glucosidase and α-amylase activity [65]. The tannin associated with the antidiabetic activity in *T. chebula* was corilagin [80]. Other components of *T. chebula*, like chebulanin, chebulagic acid and chebulinic acid, have been demonstrated to be able to inhibit the activity of maltase, an enzyme with a high activity rate in diabetic processes [81]. Furthermore, ETs and GTs isolated from *T. bellerica* and *T. chebula* have been described to improve the peroxisome proliferator-activated receptor-α and/or -γ signaling, which plays an important role in controlling the expression of genes related to the storage and mobilization of lipids, glucose metabolism, morphogenesis and inflammatory response, which have direct effects in insulin sensitivity [82].

### 2.4. Cardioprotection and Blood Circulation Improvement

The potential of polyphenols as natural antiplatelet, anti-inflammatory, and anticoagulant agents has prompted their analysis from a cardioprotective point of view. In this sense, numerous studies have demonstrated the beneficial cardiac effects of the walnut (*J. regia*). The major phenolic compounds present in *J. regia* are ETs, including HHDP derivatives which are capable of releasing EA, a well-known antioxidant related to different health benefits [46]. A work based on human aorta endothelial cells analyzed the effects of an extract from peeled fruits of *J. regia.* Initially, inflammatory processes were induced in the cultured cells by their exposure to TNF-α, which prompted the maximal expression of vascular cell adhesion protein (VCAM-1) and ICAM-1. The co-treatment of cells with *J. regia* extracts or with purified EA, one of their major components, inhibited both inflammatory biomarkers [47]. Another experiment (in vivo) utilized isoproterenol, a synthetic catecholamine capable of producing myocardium pathologies, to induce myocardial infarction. This catecholamine was administered individually or in combination with *J. regia* extracts. The presence of walnut extracts reduced the severity of the myocardial infarction in a dose-dependent manner, quantified through serum creatine kinase myocardial band- levels and the activity of lactate dehydrogenase. Additionally, the administration of *J. regia* extracts was able to reverse the negative effects of isoproterenol on oxidative markers, myocardial tissue lipids and at a histopathological level [48]. Thus, these works, among many others, demonstrated the antiatherogenic and cardioprotective potential of the consumption of *J. regia.*

Other plants have also been evaluated in terms of cardioprotective activity. For instance, extracts obtained from leaves of *R. idaeus* were evaluated through a blood ADP assay to determine how they modify blood platelet aggregation. The results showed that *R. idaeus* reduced, by more than 20%, the expression of the glycoprotein IIb/IIIa, which is involved in the reception of fibrinogen and the activation of platelets. The activation of the aggregation was nearly inhibited to 50% by the presence of the extract. When the antiplatelet activity of the extracts was tested in blood, the inhibition of platelet aggregation was reduced to less than 20% [83]. Ethanolic extracts from seeds of *Acacia senegal* were tested as antiatherosclerotic and cardioprotective agents in an in vivo experiment with rabbits subjected to a hypercholesterolemic diet. The administration of the extract reduced the levels of the total cholesterol, low- and very low-density lipoprotein (LDL and VLDL) cholesterol and triglycerides in blood. Besides, *Acacia*-treated subjects showed a lower atherogenic index accompanied by a reversion in lipid oxidation markers and histological damage [84]. Finally, bark extracts from *C. sativa* were assayed using primary cultures of neonatal rat cardiomyocytes and cardiac tissues isolated from guinea pigs. Cardiomyocytes were exposed to H_2_O_2_ to induce oxidation states, while cardiac tissues were incubated with carbachol for testing the muscarinic activity, propranolol for the adrenergic/cholinergic activities and noradrenaline for evaluating aortic muscle behavior. *C. sativa*-treated cardiomyocytes showed a dose-dependent reduction of intracellular ROS production, which directly improved cell viability. The aortic noradrenaline-induced contraction was reduced by the *C. sativa* extract, which also reduced heart rate and produced a positive inotropic effect in the left atrium/papillary (adrenergic receptor involvement was demonstrated) and negative chronotropic effect (not mediated by cholinergic receptors). Thus, the results supported the use of *C. sativa* extracts as dietary supplements since they may provide synergic beneficial effects as cardioprotective and antioxidant agents [40].

### 2.5. Wound Healing

Tannins have been demonstrated to prevent the appearance of ulcers or to accelerate wound healing, which may have different further applications. That is the case of an experiment performed with *R. imperialis*, whose anti-inflammatory and wound healing activity was investigated. In vitro assays demonstrated the antioxidant and anti-inflammatory properties and lack of cytotoxicity of the extracts, while in vivo experiments focused on their cytoprotective and healing effects, also including anti-inflammatory responses. The administration of *R. imperialis* (100 mg/kg) via gavage was shown to block the migration of neutrophils, which had a positive effect in cutaneous wounds. Besides, in vitro assays using LPS to simulate inflammation and leukocyte migration showed that the application of *R. imperialis* increased fibroblast migration up to 76% when compared with the control and reduced NO release. Additionally, the topical administration of *R. imperialis* was shown to affect collagen proliferation with a better organizational pattern than the control. In addition, in vitro experiments displayed a very low hemolysis rate for the extracts and no skin irritation potential [85]. Another approach to the wound healing strategy was presented in a work where an extract of *R. coriaria* was studied for its potential healing, anti-inflammatory and antimicrobial activities. Anti-inflammatory markers, such as the activity of myeloperoxidase and matrix metalloproteinase-8 (MMP-8) enzymes, were reduced in animals treated with *R. coriaria,* while healing indicators, such as hydroxyproline or collagen deposition, increased and the wound area was reduced, completing epithelization and scar formation by the 10th day of treatment. Different in vivo and in vitro experiments supported the antimicrobial activity of the *R. coriaria* extract. Indeed, animals with wounds infected with *Staphylococcus aureus* or *Pseudomonas aeruginosa* showed a slight delay in the healing process, reaching similar markers between the 10th and 13th day of treatment [86]. Hence, as described in this work, the healing activity of tannins exerts not just a cicatrizing effect but also an antimicrobial effect, which is crucial since wounds often get infected. The antimicrobial capacity of tannins is reviewed in the next sub-section.

### 2.6. Antimicrobial

Many works have supported the antibacterial, antifungal and antiviral properties of tannins [10,31]. For example, walnut leaves have been approved for the topical treatment of mild skin inflammation, due to their anti-inflammatory and antimicrobial activities. An ethanolic extract of immature *J. regia* fruits was able to inhibit methicillin-resistant *S. aureus* (MRSA) growth when administrated in a concentration range of 128–512 μg/mL. At 16 μg/mL, the extract reduced biofilm formation and adherence. Thus, this extract was suggested to be an efficient treatment for skin and soft tissue infection processes [87]. Similarly, extracts obtained from *S. brasiliensis* have shown antimicrobial potential against MRSA [88]. A very recent work performed transcriptome and metabolome data analysis to find the mechanism of action of tannins from *Diospyros kaki* against MRSA. The results suggest that some main mechanisms are related to physical damage of the bacterial cell membrane [89]. Tannins obtained from *A. mearnsii* were evaluated as antibacterial agents, both as free and encapsulated molecules. Free tannins showed antibacterial activity, especially against *S. aureus*, with MBC (Minimum Bactericidal Concentration) of 0.32 and 1.25 of mg/mL. They also acted against fungal (*Aspergillus niger*, MIC (Minimum Inhibitory Concentration) 0.62 mg/mL) and yeast (*Candida* sp., MIC 2.5 mg/mL) growth. The most efficient antimicrobial encapsulated showed IZs for *S. aureus, Escherichia coli, A. niger* and *Candida* sp, which were larger of those observed with the free tannins, except for *S. aureus* [34].

Several species belonging to genus *Rubus* were tested in vitro as antibacterial and antifungal agents. The main tannins involved in these activities were different among the species and plant parts analyzed. Extracts were tested against two strains of *Helicobacter pylori*, with and without the chromosomal insertion *cag,* whose presence is associated with an increased inflammatory profile. The whole extract, after 24 and 48 h of treatment, showed values of 1200 and 134 μg/mL of minimum bactericidal concentration (MBC) against the cag- *H. pylori*. Tannins and other phenolic compounds have been suggested to be involved in the antibacterial mechanism by the inhibition of bacterial ionic pumps [90]. A species from the same genus, *R. ulmifolius*, has been evaluated in terms of amoebicidal, antibacterial and antifungal activities. Trophozoites from *Acanthamoeba castellanii* showed a dose-dependent sensitivity to the extract, but not comparable with the positive control. The antibacterial and antifungal capacity of extracts was determined by inhibition zone (IZ) diameters, minimal inhibitory concentration (MIC) and MBC. *R. ulmifolius* extracts presented MIC and MBC for all bacteria and the yeast in the range of mg/mL. The best results in terms of IZ were achieved for *Escherichia coli*, *Streptococcus agalactiae* and *Candida albicans,* while Gram-positive *S. aureus* and *Enterococcus feacium* were less sensitive [91]. Furthermore, purified methanolic extracts of *R. ulmifolius*, which presented a high content of tannins (both HTs and CTs) demonstrated significant antifungal activity against five filamentous fungi: *Beauveria* sp., *Fusarium solani*, *Microsporum canis*, *Phialophora verrucosa* and *Scopulariopsis brevicaulis* [92].

Different parts of *C. sativa* (leaves, burs, outer and inner shells) were also tested for antibacterial activity. Leaves contained the highest phenolic composition, with trigalloyl-HHDP-glucose-like molecules, as major tannin representatives, also present in burs. Outer shells were rich in GA, while the main compound in the inner shell was syringetin-hexoside. Extracts from inner shells were able to inhibit the growth of the Gram-positive bacteria *Staphylococcus epidermidis, S. aureus, Enterococcus faecalis* and *E. faecium,* and Gram-negative *Klebsiella pneumoniaea* and *P. aeruginosa*, with MIC values between 25 and 50 mg/mL [41]. Other tannins present in *C. sativa*, such as vescalagin and castalagin, were isolated, purified and demonstrated to have antibacterial activity against *E. coli*. Apart from them, commercial crude extracts obtained from quebracho, chestnut and mimosa, and two classes of tannic acid and one of GA, were analyzed. From the results, it was observed that tannic acid possesses much better growth inhibitor activity than GA against *E. coli.* On the other hand, a crude extract of chestnut had stronger antibacterial properties than purified vescalagin and castalagin, probably due to the synergy exerted by the molecules contained in the extract [42]. Finally, *C. sativa* extracts have been reported to inhibit *E. coli* and *Clostridium perfringens* growth when applied at 1200 µg/mL and 3–150 µg/mL, respectively [43,93].

Tannins extracted from different genera have also been experimentally demonstrated to act as antiviral agents. For instance, an extract of *P. granatum*, with punicalagin, GA and EA as major components, was analyzed against herpes simplex virus 2. The compound punicalagin showed significant antiviral activity, comparable with the positive control. However, when used as part of the extract, the required concentration to achieve total inhibition was higher [54]. Apart from punicalagin, other work analyzed the antiviral activity of punicalin and geraniin against hepatitis B virus (HBV). When these three HTs were tested in a human hepatocyte cell line (HepG2.117), they showed a dose-dependent reduction of supernatant e antigen levels, which indicated that the tannins were interfering with the synthesis, stability or transcription of the viral DNA [53]. *Urtica dioica* and *Taraxcum officinale* exhibited inhibitory effects in the range of 126–166 μg/mL against dengue virus serotype 2 when tested in vitro using hamster kidney cells (BHK-21). Recently, an in silico evaluation of the application of 19 HTs was performed to screen their potential ability to inhibit the activity of SARS-CoV-2. Specifically, the potential allosteric ligand of different HTs with 3-chymotrypsin-like cysteine protease enzyme (described to be involved in virus transcription) was evaluated. Among the tested HTs, pedunculagin, tercatain and castalin interacted with the catalytic dyad Cys145 and His41 through stronger binding forces. Other HTs, like tellimagradin I, punicalin, chebulagic acid or β-pedunculagin, may have secondary roles in the inhibition of the activity of this catalytic target [94].

### 2.7. Other Beneficial Applications of Tannins

#### 2.7.1. Human Beings

Nowadays, there is plenty of evidence for the ethnopharmacological use of tannin-rich plants as antidiarrheal treatments. Yet, some clinical studies employing tannin extracts have shown promising results regarding the efficacy of their use and their safety. A study described how the administration of tannins reduced the duration of diarrhea caused by rotavirus in infants. Similarly, a more recent study reported that children affected by acute diarrhea presented a significant decrease in the duration of the diarrheal symptoms when administered with tannins in comparison with the standardized rehydration treatment [95,96]. These findings not only support the use of tannins as effective antidiarrheal treatments, but also provide information on their safety, since the test subjects were children. In the same way, a recent study with quebracho and chestnut extracts studied the antioxidant activity and metabolization of these extracted tannins in in vitro digestion–fermentation assays. The results evidenced the degradation of tannins by gut microbiota, producing metabolites like quercetin or sinapinic acid, as well as higher antioxidant capacity on residual solids after fermentation and increased production of short-chain fatty acids. Short-chain fatty acids are described as prebiotics in the gut [97]. This would infer that tannins also have a prebiotic effect on digestive microbiota.

A clinical trial with patients prone to developing urinary tract infections unveiled the potential of tannins as a treatment against these infections. In that case, tannins were extracted from *Serenoa repens* and orally administered as a food supplement. Although the results differed in a gender-dependent manner, leukocyte count and urinary microbiota decreased significantly in the subjects after 9 weeks, which is a relevant result, given that these patients tend to show higher levels of these markers [98].

Another uncharted potential application of tannins may be their use as anxiolytics. A recent study analyzed the performance of *T. chebula* tannin extracts on anxiety behavior, the genetic expression of gamma-aminobutyric acid (GABA) receptors and corticosterone markers, as well as in electroencephalogram assays in mice. The results appear to indicate that tannin extracts were able to ameliorate the expression of GABA receptors, selected biochemical markers and improve the oxidative status of cerebral tissue [99].

#### 2.7.2. Veterinarians

A great number of studies have evaluated the potential use of tannins and tannin supplements in livestock, such as cattle, poultry or sheep. Research has focused on their use as antimicrobials or growth promoters. Nonetheless, tannins are known antiherbivore compounds able to reduce the digestibility of proteins in said animals by their aggregation properties [31].

Legumes have been suggested as an additional ingredient to create pasture forages since they represent a protein source for livestock, improve the rumen microbiome and reduce greenhouse gas emissions. Among the range of legumes, *Lespedeza procumbens*, *Desmodium paniculatum*, *Leucaena leucocephala*, *D. ovalifolium* and *Flemingia macrophylla* possess a relatively high content of CTs. They have been suggested to modify rumen fermentation in beef cattle, probably due to the presence of tannins, so they may act similarly in animals by complexing proteins and short-chain fatty acids, which may provide a prebiotic effect, as stated [100]. Tannins have exhibited similar antidiarrheal results in cattle as those mentioned in humans. As an example, a blinded study tested the effect of a proprietary chestnut tannin extract on the duration of neonatal diarrhea in calves. The results were consistent with other previous data, notably a lowering of the duration of the diarrheic episodes without affecting the weight of the animals [45]. CTs from *L. pedunculatus* and *L. corniculatus* are reported to avoid protein degradation and to improve amino acid residue absorption at the intestinal level, which ultimately enhances animal performance, with an apparent higher ovulation rate and clean wool production in sheep [51]. Finally, tannins have also been employed to treat gastrointestinal diseases. The anthelmintic activity of tannins has been addressed, but they are also effective against protozoan parasites. This is the case of a study where *Berberis vulgaris* and *R. coriaria* extracts were applied against relevant pathogenic protozoans like *Theileria equis* and common *Babesia* species, such as *B. bovis*, *B. bigemina* or *B. caballi*, in a mouse-infected model. The experiment showed better levels of selected biomarkers in plasma, due to the synergetic effect of compounds present in the used extracts, of which tannins and flavonoids were detected as the main chemicals [101].

#### 2.7.3. Botanical

Tannins have been subjected to further research to evaluate their potential use as antifungal compounds to treat or enhance plant resistance against plant pathogens. Yet, most studies have been performed in vitro and knowledge gaps on the ecological role of tannins remain. *P. granatum* peel extracts have been tested both in vitro and in vivo against *Pseudomonas syringae*, a common and hazardous pathogen of tomato. The results demonstrated that bacterial growth was inhibited for up to 15 days after leaf inoculation [102]. In vitro antifungal assays carried out with chestnut (*C. sativa*) bur extracts, mainly composed of GA and EA moieties, as well as ETs and glycosylated flavonols, inhibited the growth and spore germination of common plant-infectious fungi *Alternaria alternata*, *Botrytis cinerea* and *Fusarium solani* [44].

#### 2.7.4. Food Additives

As previously shown, tannins have been demonstrated to be inhibitors of lipid peroxidation and scavengers of free radicals, which are the main reason for the appearance of rancid off-flavors/aromas in foods. Therefore, the utilization of tannins as a natural antioxidant in food applications is drawing attention. Among the purified tannins, the most tested is tannic acid, which has been demonstrated to reduce lipid oxidation induced by ferrous ions in a plant-based emulsion of flaxseed oil droplets. In this assay, the antioxidant activity of the tannic acid was attributed to the metal binding properties of tannins [103]. The same molecule was applied to ground chicken breast meat to determine its ability to reduce lipid and protein oxidation, maintain color and prevent rancid volatiles. The addition of 5 to 10 ppm of tannic acid improved both cooked and raw quality parameters, showing low oxidation markers, off-odor volatiles and high color parameters [104]. Extracts of tannin-rich plants such as quebracho (*S. balansae* and *S. lorentzii*) or conifers (*P. abies* or *Pinus sylvestris* L.) have also been tested as natural food preservatives with antioxidant properties to improve the shelf life of different products. Quebracho tannins (0.5–1.5%) were applied to beef patties, showing that the lowest amount was able to improve lipid stability. On the other hand, higher concentrations reduced the tenderness, softness and juiciness of meat [66]. Another study analyzed conifer tannins as antioxidants in a liposome model and in meat snacks. The tested tannins showed a high activity to prevent lipid oxidation without causing organoleptic interference in meat snacks [52]. Altogether, this research suggests that tannins, as for other polyphenols, could be added to food matrices as alternative antioxidants.

## 3. Valorization Approach and Concluding Remarks

As was mentioned before, interest in a circular economy and food or agricultural waste valorization is increasing. Therefore, biorefinery approaches for recovering bioactive molecules with target biological properties are consequently growing to face the current challenge: moving towards a circular system production model [24,105]. The use of by-products from the agri-food industry for the recovery of tannins was proposed decades ago. In 1990, it had been already suggested that pods, seeds, cake or kernel residues of some products, such as mango or cocoa, could serve as potential sources of tannins [106]. Their presence has been assessed in different by-products of the agri-food industry, such as green tea processing residues and acorn, chestnut and persimmon hulls and starch, showing antioxidant or antimicrobial properties, among others [107]. More recently, other studies have also addressed the possibility of recovering tannins from a secondary residue, such as distilled waste by-products remaining after the steam distillation of the underutilized biomass of specific trees [108]. Additionally, other experiments have been focused on assessing the bioavailability of tannins and the cellular level where they are located, i.e., they were found inside the cell lumen of parenchymatic cells and the vessels of chestnut wood [109]. Further applications are focused on obtaining other products from tannin-based sources. For instance, coffee pulp (rich in tannins) was submitted to solid state fermentation by *Penicillium verrucosum* to produce tannase [110]. However, to date, one of the biggest concerns regarding obtaining tannins is their high diversity, since this is related to their origin, extraction and purification procedures [111]. Hence, conventional and novel extraction techniques have been investigated to optimize the extraction parameters of tannin recovery from different by-products (Table 3).

Taken all together, tannins are phenolic compounds that have been used in traditional medicine, are widely distributed and have been broadly investigated for their biological properties. They can be classified according their structure and obtained mainly from vegetal sources and marine brown algae. Specific tannins of some genera or species, such as HTs, castalagin, vescalagin or punicalagin, seem to have relevance for biological activities. Important specificity differences were also observed for compounds belonging to ETs or GTs, due to the phenolic acids released after their hydrolysis (EA or GA, respectively). The most remarkable biological activities of tannins have been recorded as antioxidant, anti-inflammatory, antidiabetic, cardioprotective, healing and antimicrobial. Nevertheless, many of the indicated activities act synergically, with the antioxidant capacity being the main axis of the connection between the different biological properties. Indeed, all these interactions among biological activities are easily demonstrable, since common molecular targets are described to be involved in the different biological activities. Therefore, considering all the biological properties described in tannins obtained from natural sources, valorization could be an efficient approach to revalorize agri-food by-products. However, further study is still necessary to completely elucidate the mechanisms of action of the biological activities and improve the extraction methods and conditions to obtain tannins in an optimal way.

## Figures and Tables

**Table 1:** Examples of patented tannin applications.

Tannins	Properties	Patent No.
Punicalin, punicalagin, pedunculagin, tellimagrandin, corilagin, granatine a and b, terminalin	Treatment or prevention of cognitive and neurodegenerative disorders, metabolic syndrome, type 2 diabetes, dyslipidemia or obesity.	US20190000867A1
Punicalagins	Functional food and beverage with increased antioxidant capacity for preventing or treating hypercholesterolemia and/or hypertension	EP2033526A1
Chestnut tannins	Antioxidant or anti-microbial additive, or agent for reducing nitrosamines or mycotoxins	EP2904910B1
Ellagitannins	Treatment of bacterial infections	US20110105421A1
GA, EA, isoquercitrin, tellimagrandin I and II, pedunculagin, TGGs, PGG and di-galloyl-hexahydroxydiphenoyl-D-glucose	Inhibition or prevention of obesity, lipid storage (reducing blood triglyceride levels), hyperlipemia, arteriosclerosis and thrombosis	US7687085B2
Gallotannins and ellagitannins	Regulation of the synthesis and secretion of cytokines, including TNF-α and IL-1β	US20080070850A1
Ellagitannins	Anti-inflammatory or anti-allergic agent by the inhibition of histamine release from mast cells. Regular oral administration of product can ameliorate or prevent rhinitis, atopic dermatitis or asthma	EP0727218A3
1,3,4-tri-galloylquinic acid, galloylshikimic acid derivatives strictinin, corilagin, castalagin, vescalagin, chebulinic acid, chebulagic acid, punicalin, punicalagin, punicacortein C, cannamtannin B2	Inhibition of the propagation in human cells of a human retrovirus (HIV)	CA2001898A1
Tellimagrandin	Inhibition of Gram-positive bacteria (*Staphylococcus aureus*) growth, anti-inflammation and leukemia treatment	US8975234B2

GA: gallic acid; EA: ellagic acid; PGG: pentagalloylglucose; TGG: trigalloylglucose; AP: aerial parts, F: flowers, L: leaves, P: petals, R: roots, S: seeds, St: stems. ns: not specified.

**Table 2:** Tannin-rich genera with some representatives and their tannin chemical profile, including major compounds and their reported bioactivities.

Source	Species	Classification	Compounds	Bioactivities	Ref.
*Acacia* sp.	*A. mearnsii*	CT	Epi-FIS derivatives	Antioxidant, anti-inflammatory, antimicrobial	[33,34]
	*A. nilotica*	CT	PoGG, EA, GA, diGA, epi/gallocatechin, dicatechin derivatives	Antinociceptive, anti-inflammatory and antipyretic	[35,36]
*Castanea* sp.	*C. sativa*	HT	CAST, VES, EA, chestanin	Antioxidant, anti-inflammatory, antidiabetic, cardioprotective, antimicrobial, antifungal, antidiarrheal (vet.)	[37,38,39,40,41,42,43,44,45]
*Juglans* sp.	*J. regia*	HT	EA, pedunculagin, casuariin	Antiplatelet, cardioprotective, antiatherogenic and anti-inflammatory	[46,47,48]
*Lotus* sp.	*L. corniculatus*	CT	Heteropolymers PC: PD	Improvement of animal performance	[49,50,51]
	*L. pedunculatus*	CT			
*Picea* sp.	*P. abies*	CT	-	Antioxidant (food preservative)	[52]
*Punica* sp.	*P. granatum*	HT	Punicalagin, punicalin, geraniin	Antiviral (herpes simplex-2, hepatitis B)	[53,54]
*Quercus* sp.	*Q. robur*	HT	Castalin, vescalin, CAST, VES, GA, EA, PoGG	Antioxidant, antidiabetic	[55,56,57]
*Rhus* sp.	*R. coriaria*	CT HT	GA, QUERG, CYANG derivatives	Antimicrobial, anti-inflammatory, immunomodulatory, antiapoptotic and healing	[58,59]
*Rubus* sp	*R. fruticosus*	CT	CYANG, GA, malvidin-3-galactoside, vanillic acid	Antioxidant, anti-inflammatory, antidiabetic and gastroprotective	[60,61]
*Sargassum* sp.	*S. fusiforme*	PT	Eckol, dieckol, fuhalols	Antioxidant	[62]
	*S. muticum*	PT	PG, diphlorethol, bi- and tri-fuhalol A, B	Antioxidant, antibacterial, antiproliferative, anti-inflammatory	[63]
*Schinopsis* sp.	*S. lorentzii*	CT HT	FIS-catechin polymers TGG, PGG, quinic acid-GA esters	Antioxidant, antimicrobial, anthelmintic	[64,65,66,67]
	*S. balansae*	CT	ProFIS polymers	Antioxidant, antimicrobial, anthelmintic	[1,64,65,66,67,68,69,70,71,72,73,74,75,76,77,78,79,80,81,82,83,84,85,86,87,88,89,90,91,92,93,94,95,96,97,98,99,100,101,102,103,104,105,106,107,108]
*Terminalia* sp.	*T. chebula*	HT	Chebulinic acid, TGG	Anti-inflammatory	[68]
*Vitis* sp.	*V. vinifera*	CT	Galloylated PC, PC, PD	Antioxidant, anti-inflammatory, antiobesity	[69]

Definitions: CAST: castalagin, CT: condensed tannin, CYANG: cyanidin-3-glucoside, EA: ellagic acid, FIS: fisetinidin, GA: gallic acid, GT: gallotannin, HT: hydrolysable tannin, PC: procyanidin, PD: prodelphinidin, PG: phloroglucinol; PGG: pentagalloylglucose, PoGG: polygalloylglucose, PT: phlorotannin, QUERG: quercetin-3-glucoside, TGG: trigalloylglucose, VES: vescalagin.

**Table 3:** Examples of tannin extraction techniques from different agri-food by-products.

Species	Tannin	By-Product	Extraction Techniques	Experimental Conditions	Activity	Ref.
*Trapa quadrispinosa*	HT	Pericarps	UAE	Et/W (60/40, *v*/*v*), 30 min, 40 °C, L/S ratio 40 mL/g	Antioxidant (DPPH)	[112]
*Cupressus lusitanica* and *Cistus ladanife*	TTC	Waste distilled after steam distillation	UAE	Et S/L ratio 1:20, 30 min, 30 °C, 70% A	Antioxidant (ABTS)	[108]
Coffee *(Coffea arabica)*	Procyanidins (CT)	Pulp	UAE	W/A extract, 20 min, RT	-	[113]
Pomegranate (*P. granatum* var. *Gabsi*)	TTC	Peels	UAE	2.63 g/100g dw, 55.46% E, 30 min	Antioxidant (DPPH and ABTS)	[114]
Red grape variety (*Vitis vinifera*	CT	Pomace	HAE	NaOH, Na_2_CO_3_ or NaHCO_3_) and Na_2_SO_3_ (2.5% or 5% (*w*/*w*). S/L ratio 1:8, 120 min, 100 °C	Production of environmentally friendly wood adhesive	[115]
					Silver NPs, antimicrobial and apoptotic potential	[116]
Chestnut (*Castanea sativa*)	TTC	Shells	Maceration	Et (20 mL × 3 days × 3 times) or Et/W 7:3 *v*/*v* (20 mL × 3 days × 3 times)	Antioxidant (DPPH and TEAC)	[117]
Pomegranate (*Punica granatum* L.)	TTC	Peels	HAE	W, 2% SS and 0.5% SB, S/L ratio 1:5, 7 h, 80 ± 5 °C	-	[118]
Tea (*Camellia sinensis* L.)	TTC	Leaves	SFC-CO_2_	Supercritical CO_2_ flow rate 8 g/min, 188 bar, 50 °C, co-solvent flow rate 2.94 g/min	Antioxidant (ABTS)	[119]
*Acacia mollissima*	HT and CT	Bark	HAE and MAE	HAE: M (2h, 20 °C and 60 °C). MAE (1 min, 300W or 5 min, 150W)	-	[120]
*Myrtus communis* L.	TTC	Leaves	MAE	Et 42% (60 s, 500 W, S/L ratio 32 mL/g)	Antioxidant (DPPH, TEAC and ORAC)	[121]
*Endopleura uchi*	TTC	Bark	Maceration	Et/W 50%	Antimicrobial, cytotoxic and antioxidant	[122]
Norway spruce (*Picea abies*)	CT	Bark	Hot water extraction	10% solid content, 2% SS, 0.5% SC, (75 °C, 120 min)	-	[123]
Spruce (*Picea abies*)	TTC	Bark	SFC-CO_2_	Solvent consumption 2.5 kg CO_2_/kg product and 24.94 kg Et 70/kg product, 100 bar, 40 °C	Antioxidant (DPPH)	[124]
*Eucalyptus globulus*	EA and GA	Leaves	BMSHE	1.0 M [HO_3_S(CH_2_)4mim] HSO_4_, L/S ratio 30 mL/g. MAE: 20 min, 385 W)	-	[125]

Definitions: TTC: total tannin content, Et: ethanol, W: water, A: acetone, M: methanol, SS: sodium sulfite, SB: sodium bicarbonate, SC: sodium carbonate, RT: room temperature, DPPH: 2,2-diphenyl-1-picrylhydrazyl, TEAC: trolox equivalent antioxidant capacity, ORAC: oxygen radical absorbance capacity, ABTS: 2,2′-azino-bis(3-ethylbenzothiazoline-6-sulfonic acid, UAE: ultrasound-assisted extraction, HAE: heat-assisted extraction, MAE: microwave-assisted extraction, SFC-CO_2_: supercritical carbon dioxide extraction, BMSHE: Brønsted acidic ionic liquid-based microwave-assisted simultaneous hydrolysis and extraction.

## Data Availability

Not applicable.

## Abbreviations

**Tannins**	
CoT	Complex tannin
CT	Condensed tannin
EA	Ellagic acid
ET	Ellagitannin
GA	Gallic acid
GT	Gallotannin
HHDP	Hexahydroxydiphenol
HT	Hydrolysable tannin
PC	Procyanidin
PD	Prodelphinidin
PG	Phloroglucinol
PGG	Pentagalloylglucose
PoGG	Polygalloylglucose
PT	Phlorotannin
TGG	Trigalloylglucose
**Bioactivities and Assays**	
ADP	Adenosine diphosphate
Bcl-2	Apoptosis inhibitor gen
CAS	Caspase
CAT	Catalase
COX	Cyclooxygenase
CRP	C-reactive protein
DM	Diabetes mellitus
DNA	Deoxyribonucleic acid
GABA	Gamma-aminobutyric acid
GSH	Glutathione (reduced)
HBeAG	E antigen of the hepatitis B virus
HBV	Hepatitis B virus
HIV	Human immunodeficiency virus
ICAM	Intercellular adhesion molecule
IL	Interleukin
iNOS	Nitric oxide synthase
IZ	Inhibition zone
JNK	C-Jun N-terminal kinase
LPS	Lipopolysaccharide
MAD	Malondialdehyde
MBC	Minimum bactericidal concentration
MIC	Minimal inhibitory concentration
MMP	Matrix metalloproteinase
mRNA	Messenger ribonucleic acid
MRSA	Methicillin-resistant *Staphylococcus aureus*
NADPH	Nicotinamide adenine dinucleotide (reduced)
NF-κB	Nuclear factor-κB
NO	Nitric oxide
NSAID	Nonsteroidal anti-inflammatory drug
RNA	Ribonucleic acid
ROS	Reactive oxygen species
SOD	Superoxide dismutase
TNF-α	Tumor necrosis factor-α
VCAM	Vascular cell adhesion protein
VEGF	Vascular endothelial growth factor
